# Graphical Tools for Network Meta-Analysis in STATA

**DOI:** 10.1371/journal.pone.0076654

**Published:** 2013-10-03

**Authors:** Anna Chaimani, Julian P. T. Higgins, Dimitris Mavridis, Panagiota Spyridonos, Georgia Salanti

**Affiliations:** 1 Department of Hygiene and Epidemiology, School of Medicine, University of Ioannina, Ioannina, Greece; 2 School of Social and Community Medicine, University of Bristol, Bristol, United Kingdom; 3 Centre for Reviews and Dissemination, University of York, York, United Kingdom; 4 Department of Primary Education, University of Ioannina, Ioannina, Greece; 5 Department of Medical Physics, School of Medicine, University of Ioannina, Ioannina, Greece; Institut de recherches cliniques de Montréal (IRCM), Canada

## Abstract

Network meta-analysis synthesizes direct and indirect evidence in a network of trials that compare multiple interventions and has the potential to rank the competing treatments according to the studied outcome. Despite its usefulness network meta-analysis is often criticized for its complexity and for being accessible only to researchers with strong statistical and computational skills. The evaluation of the underlying model assumptions, the statistical technicalities and presentation of the results in a concise and understandable way are all challenging aspects in the network meta-analysis methodology. In this paper we aim to make the methodology accessible to non-statisticians by presenting and explaining a series of graphical tools via worked examples. To this end, we provide a set of STATA routines that can be easily employed to present the evidence base, evaluate the assumptions, fit the network meta-analysis model and interpret its results.

## Introduction

Network meta-analysis (NMA) synthesizes data from a network of trials about more than two competing healthcare interventions. The integration of direct evidence (from studies directly comparing interventions) with indirect evidence (information about two treatments derived via a common comparator) increases the precision in the estimates and produces a relative ranking of all treatments for the studied outcome [1], [2]. The assumption of consistency (agreement between direct and indirect sources of evidence) underlies the methodology and if it holds, NMA can give valuable information to patients, practitioners and decision makers [3], [4].

The advantages of NMA have made it an increasingly popular method in comparative effectiveness research. However, NMA remains, to a large degree, a privilege for researchers with advanced computational and statistical knowledge. It has been criticized for its complexity, for involving assumptions that are difficult to evaluate and for producing outputs that cannot be easily understood and interpreted by clinicians. The lack of a user-friendly implementation framework with tools to evaluate the assumptions of the analysis and present the results has contributed to this criticism.

Graphical tools can provide comprehensive and easily understandable ways to present results of statistical analyses, particularly when a large amount of data is involved [5]. Various plots have been suggested for summarizing the evidence from studies on the relative effectiveness or acceptability of two interventions [6], [7]. Many of these tools are difficult to apply to NMA without modifications. For example, forest plots facilitate the inspection of the evidence base and its characteristics but may be less informative when several comparisons are present. Heterogeneity and its impact on summary estimates are also difficult to display graphically – or even numerically – in an understandable way. Funnel plots to identify the presence of small-study effects provide further challenges in a NMA context because observed effect sizes refer to different treatment comparisons.

Valid results from NMA depend on the evidence network being internally consistent: direct and various sources of indirect evidence should be in agreement. Graphical tools may help investigators to spot parts of the evidence network that appear inconsistent, or to inform judgments about the plausibility of consistency [8]. Finally, NMA results are not easy to interpret and do not always facilitate decision-making [9], [10]. As the number of competing treatments included in a networks of interventions increases, the need for a concise and informative presentation of results becomes more important.

A recent update in the multivariate meta-analysis routine in STATA (mvmeta command) makes NMA possible within one of the most widely used software for meta-analysis, and we expect that this will popularize the method [11]. With this important development as our starting point, we introduce a suite of STATA routines to evaluate the assumptions and graphically present NMA results. We created STATA routines that extend existing graphical tools used in pairwise meta-analysis and we also developed new tools specifically for NMA. Emphasis is placed on practical issues of applying and presenting NMA. A thorough review of statistical methodology for NMA has been described previously [12], [13].

We first present in Section 2 three working examples that will be used to present the developed STATA routines. The structure of the rest of the paper follows a typical analysis of a network of randomized controlled trials. In Section 3 we discuss graphs that enhance understanding of the dataset and facilitate the visualization of the evidence with respect to its characteristics. Section 4 briefly summarizes how to fit a NMA model using mvmeta in STATA. In Section 5 we describe graphical and numerical ways to display important assumptions of the joint analysis. Finally, Section 6 focuses on suggestions for graphical and numerical presentation of the results. We provide the script files for the full analysis in the Appendix S1.

## Materials and Methods

### 1 Examples of network meta-analyses and STATA routines

To enhance interpretation of all presented graphical and numerical summaries we used three worked examples of NMAs. The first example compares 14 antimanic drugs for acute mania [14]. The network included 47 studies reporting on efficacy (measured as the number of responders out of total randomized) and 64 studies reporting on acceptability (measured as the number of dropouts out of total randomized). The second example is a network of 62 studies that evaluate the effectiveness of four different percutaneous coronary interventions for non-acute coronary artery disease [15]. The third example is a network of 27 studies forming a star-shaped network (i.e. all active treatments are compared only with placebo) that evaluated the effectiveness of six biologic agents for rheumatoid arthritis [16]. The outcome in this network was benefit from treatment defined as a 50% improvement in patient- and physician-reported criteria of the American College of Rheumatology (ACR50). The datasets and the STATA routines can be found online in www.mtm.uoi.gr and more detail is provided in the Appendix S1. To be able to carry out the analysis described below, version 3.01 (or later) of the command metan, version 2.6.1 (or later) of metareg and version 2.5.5 (or later) of mvmeta are required.

### 2 Presenting the evidence base

#### 2.1 Network plot

The plot of a network of interventions is a visual representation of the evidence base and offers a concise description of its characteristics. It consists of nodes representing the interventions being compared and edges representing the available direct comparisons (comparisons evaluated in at least one study) between pairs of interventions.

The amount of available information can be presented by ‘weighting’ the nodes and edges using different node sizes and line thicknesses. For instance, each treatment node or each comparison edge can be weighted according to the number of studies including either that treatment or that comparison. This illustrates which interventions are more frequently compared. Node weighting according to other intervention-specific characteristics, such as market price, may be useful depending on the review research questions.

Rather than weighting edges by amount of information, the use of weighted edges according to the distribution of study-specific variables may assist in evaluation of the transitivity assumption [17], [12]. Transitivity in a network implies that the available treatment comparisons do not differ with respect to the distribution of effect modifiers. Adjusting the width of each edge to be proportional to a continuous effect modifier (e.g. year of publication, baseline risk) and visual inspection of the comparability of the comparisons collated in the network can be a useful aid in deciding whether the transitivity assumption is likely to hold.

We developed a STATA command, called networkplot, to produce plots of the evidence base. Let t1 and t2 be two variables that include the codes or names of the two treatments being compared in each available direct comparison then typing

. networkplot t1 t2

gives a plot with both nodes and edges weighted according to the number of studies involved in each direct comparison. To specify alternative weighting variables the options nodeweight() and edgeweight() can be used.

**Figure 1:**
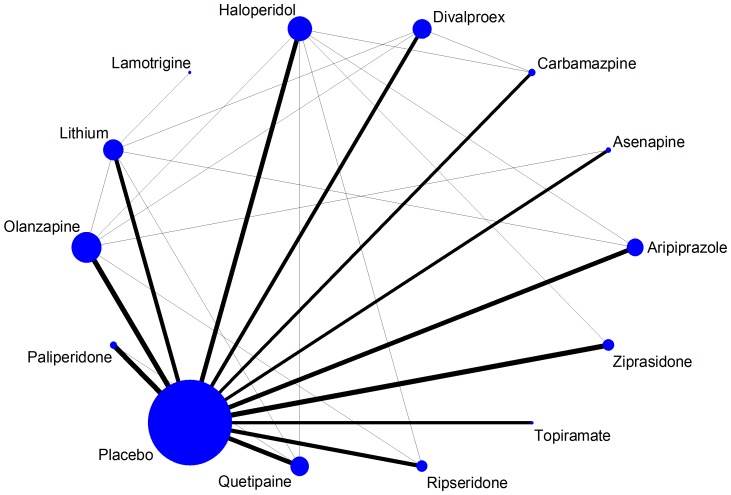
Network plot of the acute mania network (efficacy outcome). Nodes are weighted according to the number of studies including the respective interventions. Edges are weighted according to the mean control group risk for comparisons between placebo and active treatment. Edges connecting two active treatments have been given minimal weight.

In Figure 1 we plot the acute mania network for the efficacy outcome. The size of nodes shows that placebo is the most frequent comparator across the studies. Baseline risk might be an important effect modifier in this synthesis, so we weight all edges connecting placebo with an active treatment according to the mean response rate in the placebo arms. The mean placebo response rate ranged between 22% and 38% across the 12 placebo-controlled comparisons; no important differences in the width of the edges can be seen.

**Figure 2:**
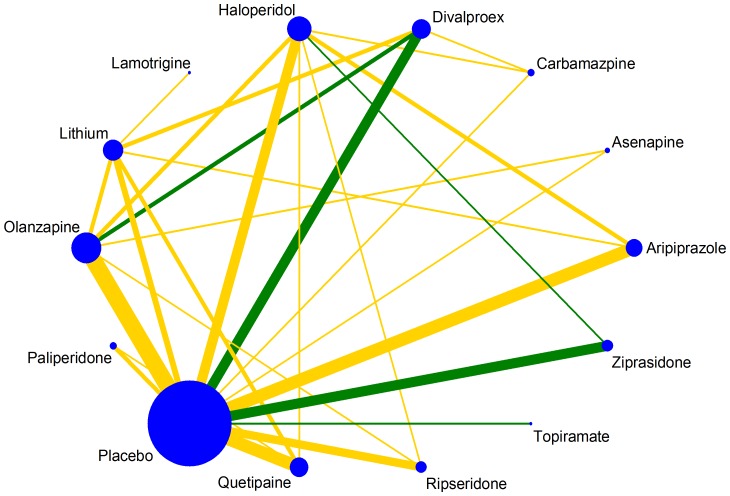
Plot of the acute mania network (efficacy outcome) using coloured edges according to adequacy of allocation concealment estimated as the level of bias in the majority of the trials and weighted according to the number of studies in each comparison.

A particularly important comparison-level characteristic is the quality of the studies. Trials with design limitations may lead to biased summary treatment effect estimates when included in a meta-analysis. To present the risk of bias for each direct comparison in the network, colored edges can be employed. Green, yellow and red colors are being used to denote pairwise meta-analyses of low, unclear and high risk of bias. For instance, inadequate allocation concealment was considered an important source of bias in the acute mania network (Figure 2). There are four comparisons at low risk of bias and none at high risk. The option edgecolor() in networkplot command allows for colored edges; the default is according to a three-level study-specific variable while more than three levels are allowed with user-specified colors. A variable called bias that contains scores for each study according to a particular bias component (low, unclear or high or coded as 1, 2, 3 respectively) should be specified in the data. Then the command

. networkplot t1 t2, edgecolor(by bias)

produces a network plot where the comparison-specific bias level has been estimated as the bias level in the majority of included studies in each comparison. A suboption allows specification of alternative ways to summarize the study-specific scores and obtain comparison-specific bias judgments (e.g. by bias takes the average bias score; see the help file for more details).

#### 2.2 Contribution plot: presenting the influence of each direct piece of evidence

Each direct comparison in NMA contributes differently to the estimation of the network summary effects. It is sometimes useful to identify the most influential comparisons for each network estimate and for the entire network. For instance, a comparison with high risk of bias but low contribution to the network estimates may not be an important threat to the validity of the NMA results. The weight that each direct comparison has is a combination of the variance of the direct treatment effect and the network structure [8], [18]. Comparisons with much direct information are highly influential on their ‘neighboring’ comparisons (e.g. comparisons in the same first order loop). On the other hand, comparisons for which little direct evidence exists are less influential for the rest of the network and benefit the most of it. In other words, the less influential parts of the network are those that benefit most from the network. Important differences in the variance of direct estimates between comparisons can affect their relative contribution to the other comparisons [18], [19]. The percentage contribution of each direct comparison to each network estimate can be summarized in a matrix with columns and rows corresponding to the direct and network estimates respectively.

**Figure 3:**
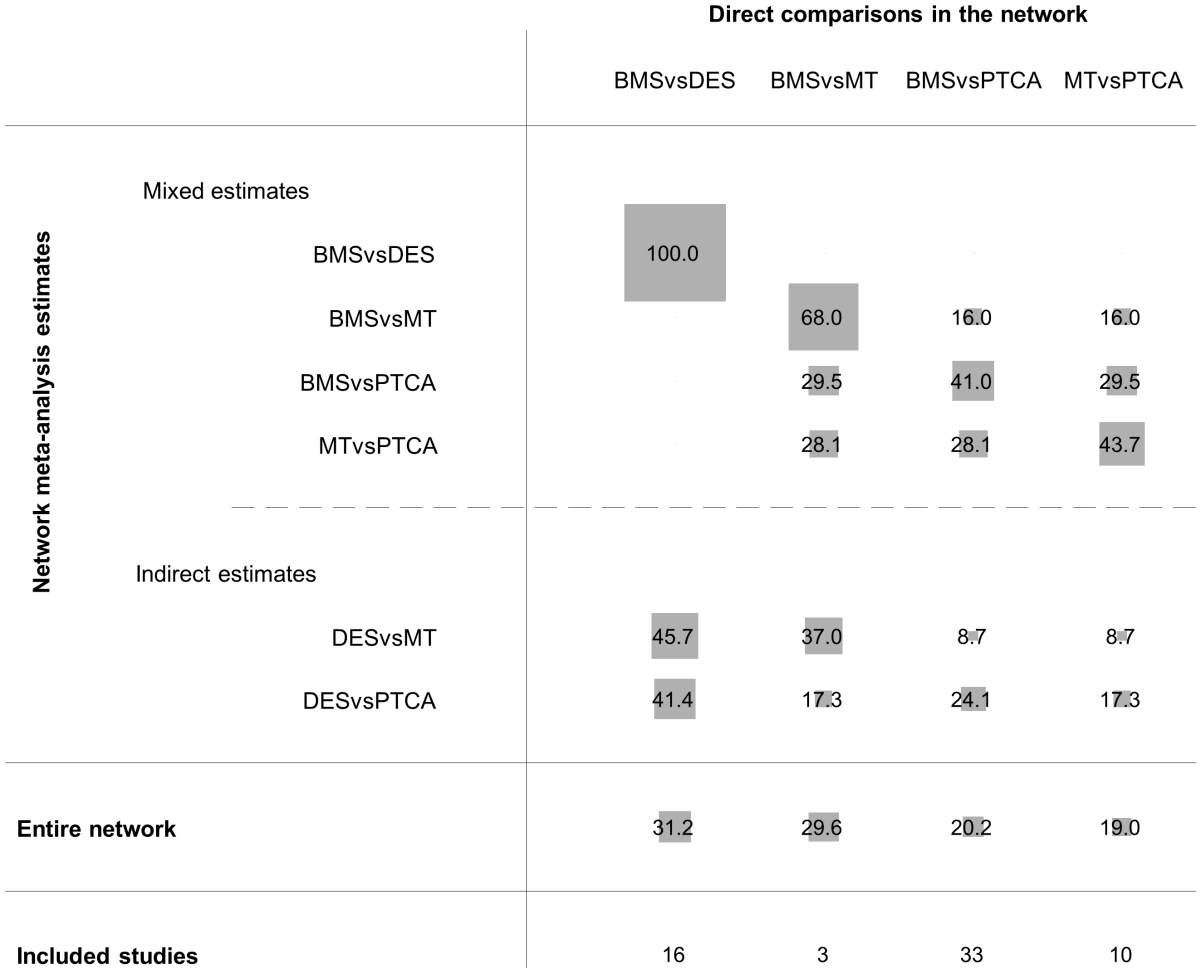
Contribution plot for the coronary artery disease network. The size of each square is proportional to the weight attached to each direct summary effect (horizontal axis) for the estimation of each network summary effects (vertical axis). The numbers re-express the weights as percentages. (MT =  medical therapy, PTCA =  percutaneous transluminal balloon coronary angioplasty, BMS =  bare-metal stents, DES =  drug-eluting stents).

Figure S1 presents the coronary artery disease network and Figure 3 presents the contribution plot for each direct comparison. The columns represent the four observed direct comparisons and the rows represent all possible pairwise comparisons. These are informed by direct evidence alone (BMS vs. DES), by mixed evidence (BMS vs. MT, BMS vs. PTCA and MT vs. PTCA) or by indirect evidence alone (MT vs. DES and DES vs. PTCA). The contribution of each direct comparison is presented by using weighted squares along with the respective percentages. For example, because there is no indirect evidence for BMS vs. DES, 100% of the information comes from the direct evidence. The NMA estimate for the DES vs. MT comparison is informed (indirectly) by all four direct comparisons with contributions 45.7%, 37%, 8.7% and 8.7%. Thus the direct comparisons BMS vs. DES and BMS vs. MT are the most influential when comparing indirectly DES to MT. The most informative direct evidence in the network is BMS vs. DES with an overall contribution of 31.2% to the network estimates.

We developed the STATA command netweight to produce the contribution plot. The netweight command needs four arguments; the study-specific effect sizes (e.g. lnOR) for each observed direct comparison, their standard errors (e.g. selnOR) and the treatments being compared (e.g. t1 t2 assuming that all effect sizes have been calculated as t1 vs. t2). The syntax to derive the plot is

. netweight lnOR selnOR t1 t2

Note that the direct estimates are derived using a comparison-specific random effects model; if a comparison is informed by less than two studies a fixed effects model is employed. However, in NMA we often assume a common heterogeneity parameter across comparisons. If the heterogeneity value is known (e.g. estimated from the mvmeta command), this can be taken as an argument in netweight command and will be used in the estimation of all pairwise direct treatment effects.

### 3 Performing network meta-analysis

A popular estimation of the NMA model is via a meta-regression routine such as metareg in STATA. The model uses as covariates the basic parameters (a set of comparisons sufficient to generate all possible comparisons via the consistency equations) and assumes that heterogeneity is independent of the comparison being made [20]. However, the current meta-regression routines fail to model properly the correlations induced by treatment effects estimated in multi-arm trials. Thus, in the presence of multi-arm trials, researchers have often preferred to perform NMA in a Bayesian framework that offers more flexibility [21], [22]. White et al. and Higgins et al. recently described NMA as a multivariate meta-analysis model [23], [24]. A major update in the standard STATA command mvmeta makes NMA possible within a frequentist setting and properly accounts for correlations between effect sizes from multi-arm studies [25]. Below, we briefly discuss the use of mvmeta in STATA to obtain NMA estimates and we refer to the original paper for more details [25].

In a network with *T* treatments, the model assumes that there is a reference treatment *A* present in all studies and 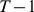 basic parameters-‘outcomes’ formed by all contrasts *AX* with 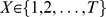
(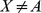). A simple data imputation technique is employed for studies that do not report treatment *A* that imputes minimal information for the missing reference arm [23], [25].

Consider for example the network of the interventions for rheumatoid arthritis; the six basic parameters are the comparisons of all active treatments versus placebo. The input in mvmeta consists of all study-specific yAX pairwise effect sizes (in our example the log-odds ratios), their respective variances SXX and covariances SXY for all studies. A covariance is calculated only for studies reporting more than two arms and is equal to the variance of the common arm between comparisons (i.e. the reference arm 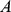). Once all variables yAX, SXX and SXY have been calculated, typing

. mvmeta y S

gives the network meta-analysis estimates for all basic *AX* comparisons. All other relative treatment effects can be derived using the consistency equations. These can be derived using the command lincom. For example, the network treatment effect between two active treatments *XY* and its standard error can be obtained by typing

. lincom yAY-yAX

The heterogeneity in the model can be assumed to be equal across comparisons or different (options bscov(proportional matexp) and bscov(unstructured) respectively).

### 4 Evaluating and presenting assumptions of the NMA

#### 4.1 Inconsistency plot

Inconsistency refers to differences between direct and various indirect effect estimates for the same comparison [26]. Important inconsistency threatens the validity of the results and if present, needs further exploration to identify possible sources of disagreement. Several approaches have been developed to deal with inconsistency in a network of interventions [26]–[29]. A simple and easy method to apply is to look at each closed loop in the network. We consider only triangular (formed by three treatments all compared with each other) and quadratic (formed by four treatments that each one is compared exactly with two other treatments in the loop) loops. If a quadratic loop can be decomposed into two (nested) triangular loops, we consider only the latter. In each loop we estimate the inconsistency factor (*IF*) as the absolute difference between direct and indirect estimates for one of the comparisons in the loop. We can also derive a 95% confidence interval (CI) and a z-test for *IF* [17], [29]. Not that *IF* is the logarithm of the ratio of two odds ratios (*RoR*) from direct and indirect evidence in the loop; *RoR* values close to 1 mean that the two sources are in agreement. As statistical power of the z-test is expected to be low, the CI of the inconsistency *RoR* should be examined and if it includes large values, further investigation is needed to identify possible sources of inconsistency.

To make inferences about inconsistency in a network of interventions, which usually includes many closed loops, we need to evaluate the inconsistency *RoR*s in every loop. All *RoR*s with their 95% CI can be jointly presented in a forest plot. Our command ifplot identifies all triangular and quadratic loops in a network, estimates inconsistency and plots the absolute *IF* values and confidence intervals (which are truncated at zero since the direction of the *IF* is unimportant). Loops are ordered according to the magnitude of the point estimate *IF*s (from larger to smaller). The syntax is

. ifplot lnOR selnOR t1 t2 id

where id is a variable identifying the studies. The option eform can be added to plot *RoR*s instead of the *IF*s.

**Figure 4:**
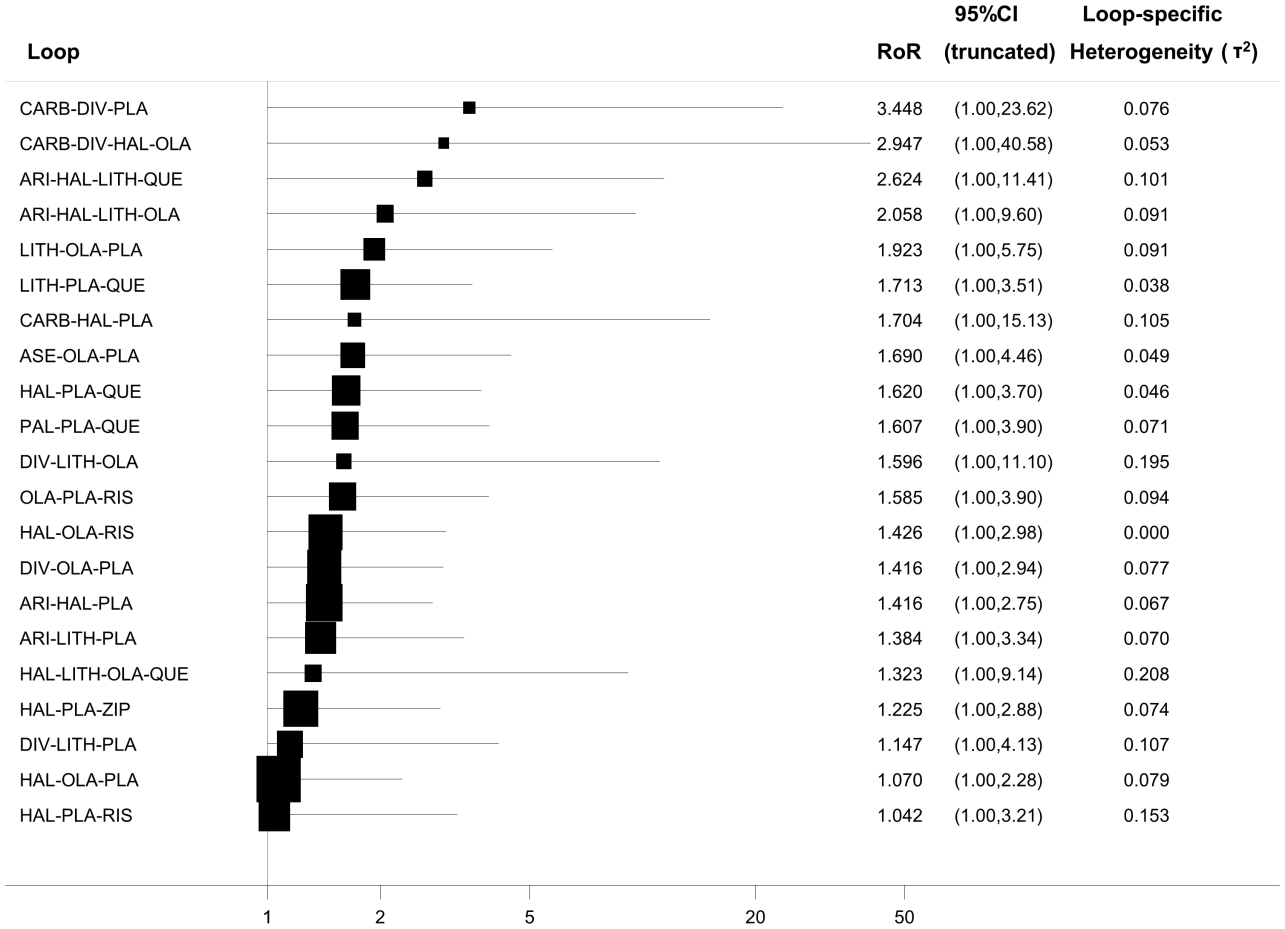
Inconsistency plot for the acute mania network (for the efficacy outcome) assuming loop-specific heterogeneity estimates using the method of moments estimator. (PLA =  placebo, ARI =  aripiprazole, ASE =  asenapine, CARB =  carbamazpine, DIV =  divalproex, HAL =  haloperidol, LAM =  lamotrigine, LITH =  lithium, OLA =  olanzapine, QUE =  quetipaine, RIS =  risperidone, TOP =  topiramate, ZIP =  ziprasidone, PAL =  paliperidone).

In Figure 4 we illustrate the inconsistency in the loops of the acute mania network (efficacy outcome) assuming a common loop-specific heterogeneity variance estimated using the method of moments. The plot shows that in a total of 21 loops there is none with statistically significant inconsistency as all confidence intervals for *RoR*s are compatible with zero inconsistency (*RoR = *1). However, several of the loops include values of high inconsistency (e.g. mean *RoR* larger than 2) meaning that the direct estimate can be twice as large as the indirect estimate or the opposite (the indirect estimate is twice the direct). For those loops we cannot draw a safe conclusion regarding the presence or not of inconsistency. Whereas this approach is easy to implement, the results require careful interpretation because of the presence of many underpowered and correlated tests. The absence of statistically significant inconsistency is not evidence against the presence of inconsistency [12].

It has been shown that different estimators of the heterogeneity parameter and the various assumptions about the similarity of heterogeneity across comparisons can lead to different conclusions about the presence of statistical inconsistency [30]. Hence we allow for three different scenarios about heterogeneity: that each comparison has a different heterogeneity parameter, that all comparisons within a loop share a common heterogeneity variance and finally that all comparisons in the network share a common heterogeneity variance. The option tau2() in ifplot command allows the choice between these options: tau2(comparison) for comparison-specific heterogeneity, tau2(loop) for loop-specific heterogeneity (estimated via meta-regression in the loop) and tau2(#) to impute a specific value for the heterogeneity variance (which would typically be obtained from results of a NMA that assumes a common heterogeneity variance).

Numerous methods are available to estimate the heterogeneity variance in meta-analysis [31]. Method of moments and restricted maximum likelihood are those most commonly used and have been implemented in STATA's metan command along with an empirical Bayes method [32], [33]. To change between these three estimators in ifplot the options mm, reml and eb are available for the loop-specific heterogeneity approach.

The mvmeta command allows us to employ alternative approaches to evaluate inconsistency [26], [27]. It can incorporate two types of inconsistency: differences between direct and indirect estimates and differences between trials with different designs (e.g. two-arm vs. multi-arm).

#### 4.2 ‘Comparison-adjusted’ funnel plot

A funnel plot is a scatterplot of the study effect size versus some measure of its precision, often its inverted standard error. It is the most common tool used to assess the presence of small-study effects in a meta-analysis [34]. A funnel plot which is asymmetrical with respect to the line of the summary effect implies that there are differences between the estimates derived from small and large studies.

Extending the use of funnel plots into network meta-analysis needs to account for the fact that studies estimate effects for different comparisons. As a result, there is not a single reference line against which symmetry can be judged. To account for the fact that each set of studies estimates a different summary effect we suggest the ‘comparison-adjusted’ funnel plot. Before using this plot, investigators should order the treatments in a meaningful way and make assumptions about how small studies differ from large ones. For example, if they anticipate that newer treatments are favored in small trials, then they could name the treatments from oldest to newest so that all comparisons refer to ‘old versus new intervention’. Other possibilities include defining the comparisons so that all refer to an active treatment versus placebo or sponsored versus non-sponsored intervention.

In the ‘comparison-adjusted’ funnel plot the horizontal axis presents the difference between the study-specific effect sizes from the corresponding comparison-specific summary effect [35]. For example, in a triangle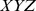, we get the three direct summary estimates 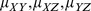
from simple pairwise meta-analyses. The treatments have been named, say, from the oldest to newest. Then, for studies that compare treatments 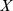 and 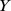 (providing and observed effect 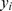) the horizontal axis represents the difference 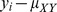. Similarly, it represents 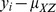 and 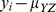 for studies comparing *XZ* and *YZ* respectively. In the absence of small study effects the ‘comparison-adjusted’ funnel plot should be symmetric around the zero line.

To produce a comparison-adjusted funnel plot in STATA our command netfunnel can be used:

. netfunnel lnOR selnOR t1 t2, bycomparison

(assuming that effect size lnOR has been estimated as t1 vs. t2)

The option bycomparison adds comparison-specific colors to the studies.

The routine netfunnel plots the comparisons as ‘treatment alphabetically or numerically earlier versus later treatment' (e.g. A vs. B or 1 vs. 2) for string or numerical treatment identifiers. Therefore, missing (small) studies lying on the right side of zero line suggest that small studies tend to exaggerate the effectiveness of treatments named earlier in alphabet compared to those later for a harmful outcome. If the outcome is beneficial such asymmetry would indicate that small-study effects favor treatments later in the alphabetical or numerical order. A ‘comparison-adjusted’ funnel plot is meaningless unless the treatments are named in an order that represents a characteristic potentially associated with small study effects. Consequently, we recommend its use only when specific assumptions about the directions of small study effects can be made.

**Figure 5:**
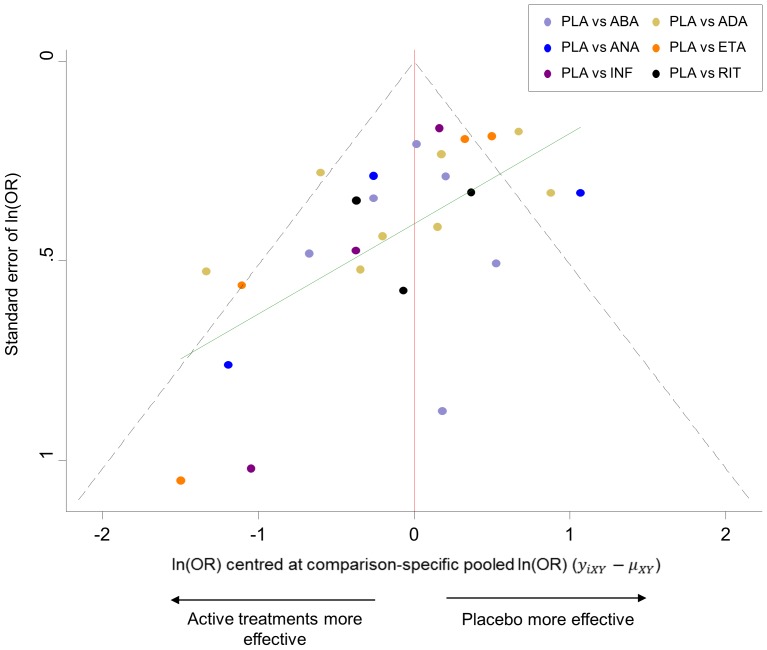
Comparison-adjusted funnel plot for the rheumatoid arthritis network. The red line represents the null hypothesis that the study-specific effect sizes do not differ from the respective comparison-specific pooled effect estimates. The green line is the regression line. Different colours correspond to different comparisons.

Figure 5 shows the funnel plot for the rheumatoid arthritis network which provides an indication for the presence of small-study effects. The plot indicates that small studies tend to show that the active treatments are more effective than their respective comparison-specific weighted average effect.

The options fixed and random in netfunnel command specify whether the summaries will be derived from a fixed- or random-effects model. A linear regression line of the comparison-specific differences 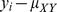 on the standard error of 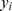 can be fitted to the plot using the addplot option, e.g. addplot(lfit selnOR _ES_CEN) (see the green line in Figure 5).

After running netfunnel a new variable is added to the dataset named _ES_CEN that includes the differences between study-specific effect sizes and comparison-specific summary estimates.

As with the conventional funnel plot, caution is needed in interpretation. Asymmetry should not be interpreted as evidence of publication bias. If the funnel plot suggests the presence of small-study effects, investigators can explore this further by employing appropriate network meta-regression or selection models [36], [37].

#### 4.3 Predictive intervals plot

Heterogeneity is an important feature in both pairwise and network meta-analysis. In pairwise meta-analysis, visual inspection of the forest plot, the I^2^ measure, the variance of the distribution of random effects 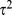
and its 95% confidence intervals, or the Q-test are used to infer about the magnitude of heterogeneity and place the summary effect into context. In NMA, the between-studies variance 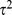 often assumed to be common across comparisons, is typically used to present heterogeneity across the network. Although multivariate heterogeneity measures such as 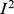 for multivariate meta-analysis have been developed [38], they have not been applied yet to NMA. Note that in NMA we often assume a common heterogeneity variance across all pairwise comparisons. Some comparisons can be affected more than others by the magnitude of the common heterogeneity variance estimate regarding the amount of additional uncertainty anticipated in future studies. We suggest the presentation of NMA mean summary effects together with their predictive intervals to facilitate interpretation of the results in the light of the magnitude of heterogeneity.

Predictive intervals (PrI) provide an interval within which the estimate of a future study is expected to be [39], [40]. They are computed as 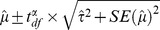 where 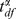 is the 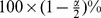 percentile of the *t*-distribution with 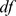
degrees of freedom (in NMA we suggest this is set to number of studies – number of comparisons with data – 1 [41]) and 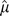 is the meta-analysis summary effect.

**Figure 6:**
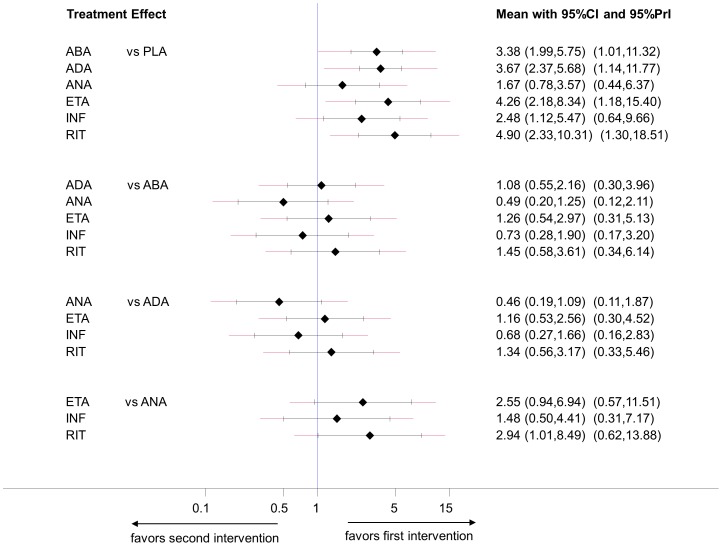
Predictive interval plot for the rheumatoid arthritis network on a logarithmic scale. The black solid lines represent the confidence intervals for summary odds ratios for each comparison and the red dashed lines the respective predictive intervals. The blue line is the line of no effect (odds ratio equal to 1).

A forest plot of the estimated summary effects along with their confidence intervals and their corresponding PrI for all comparisons summarizes in one plot the relative mean effects, predictions and the impact of heterogeneity on each comparison. Such a plot is presented in Figure 6 for the biologics in rheumatoid arthritis. The estimated common between-study variance was 0.26 and all six active treatments appear more effective than placebo. The plot indicates that for only one of these comparisons (infliximab vs. placebo) the PrI is wide enough compared with the CI to suggest that in a future study the active treatment can appear less effective than placebo, although the lower CI limit does not cross the line of no effect.

Such a plot can be produced using our STATA command intervalplot after running the mvmeta command as follows:

. intervalplot, mvmetaresults

For dichotomous outcomes the option eform can be added to plot the estimates on the odds ratio or risk ratio scale (instead of their logarithms).

### 5 Presenting the results

#### 5.1 Ranking plots for a single outcome using probabilities

One of the advantages of network meta-analysis is that it can provide information about the ranking of all evaluated interventions for the studied outcome [2], [42]. Probabilities are often estimated for a treatment being ranked at a specific place (first, second, etc.) according to the outcome.

Ranking of treatments based solely on the probability for each treatment of being the best should be avoided. This is because the probability of being the best does not account for the uncertainty in the relative treatment effects and can spuriously give higher ranks to treatments for which little evidence is available. So-called rankograms and cumulative ranking probability plots have been suggested as a reliable and comprehensive graphical way to present ranking probabilities and their uncertainty [2]. A rankogram for a specific treatment *j* is a plot of the probabilities of assuming each of the possible *T* ranks (where *T* is the total number of treatments in the network). The cumulative rankograms present the probabilities that a treatment would be among the *n* best treatments, where *n* ranges from one to *T*. The surface under the cumulative ranking curve (SUCRA), a simple transformation of the mean rank, is used to provide a hierarchy of the treatments and accounts both for the location and the variance of all relative treatment effects [2]. The larger the SUCRA value, the better the rank of the treatment.

The mvmeta command can provide ranking probabilities using the option pbest(min|max, all zero). Options min or max specify whether larger or smaller treatment effects define a better treatment, while all and zero specify the estimation of probabilities for all possible ranks including the reference treatment. The estimated probabilities can be stored as additional variables in the dataset by adding the suboption gen()in pbest() and predictive ranking probabilities (the probability that each treatment will be placed in each rank in a future study [39], [40]) can be estimated with the suboption predict.

Our STATA command sucra produces rankograms and computes SUCRA values using the ranking probabilities (e.g. as estimated with the mvmeta) as input. If prob1 prob2 etc, is a list of variables including all ranking probabilities (one variable per treatment for each possible rank) as derived from mvmeta then typing

. sucra prob*,mvmetaresults

plots the cumulative rankograms for all treatments.

**Figure 7:**
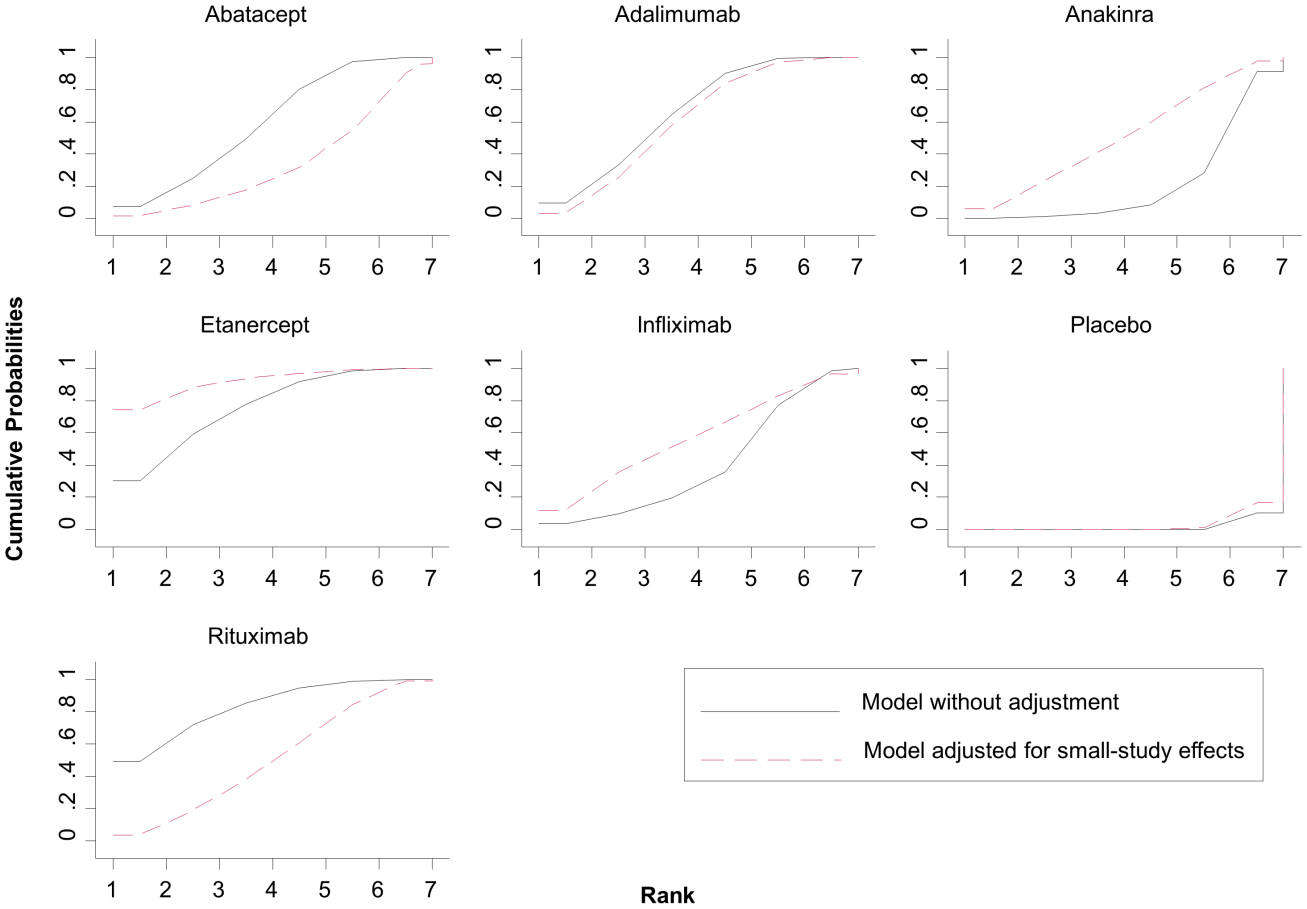
Plots of the surface under the cumulative ranking curves for all treatments in the rheumatoid arthritis network. Black solid lines correspond to the unadjusted model and red dashed lines to the adjusted for small-study effects model.

In Figure 7 we present cumulative rankograms for the network of rheumatoid arthritis trials. The SUCRA values provide the hierarchy for the six active treatments; 1.8%, 59.9%, 66.2%, 21.8%, 75.9%, 41%, 83.4% for placebo, abatacept, adalimumab, anakinra, etanercept, infliximab, rituximab respectively. The cumulative rankograms can also be used to compare different models. In Figure 7 we present also the results from a network meta-regression accounting for small-study effects (using the variance of the log-odds ratios as covariate). The graph shows that small-study effects materially alter the relative effectiveness and ranking of treatments and adjustment will put etanercept and anakira in more favourable order compared with rituximab and abatacept respectively. The option compare() in the command sucra can be used to compare two ranking curves.

#### 5.2 Ranking plot for a single outcome using multidimensional scaling

An alternative approach to rank the competing treatments is by using multidimensional scaling (MDS) [43], which was recently employed to examine the inconsistency in a network of interventions [44]. Multidimensional scaling is a family of multivariate techniques for the analysis of proximity data on a set of stimuli aiming to reveal the latent structure underlying the data and to represent them spatially or geometrically, preferably on a system of coordinate axes.

The input to MDS is a square, symmetric matrix indicating either similarities or dissimilarities among objects. We consider the *T* treatments of a network to be the objects. We run a NMA model (e.g. using mvmeta) to obtain the network estimates 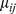 and their standard errors for every possible pair of treatments 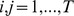
with 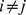. The absolute value of 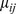 defines the dissimilarity between treatments *i* and *j*. To apply MDS for treatment ranking, we create a symmetric 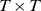
matrix of dissimilarities where dissimilarity for the 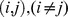 object is defined as the absolute value of the effect size (estimated from NMA) when treatment 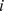 is compared to treatment *j* and zero for 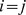. To ensure that all values in the matrix share a common distribution we weight the absolute effect sizes by their inverse standard errors or variances. The purpose of MDS is to transform treatment effects into distances represented in multidimensional space. More specifically, MDS uses stress majorization to find the set of distances in a *p*-dimensional space (usually 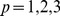 so that it can be represented graphically) that are as close as possible to the observed dissimilarities. Here, we assume that the rank of treatments is the only dimension underlying the outcome. This leads to a dimension reduction from the 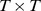
matrix to a 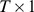 vector representing treatment order.

**Figure 8:**
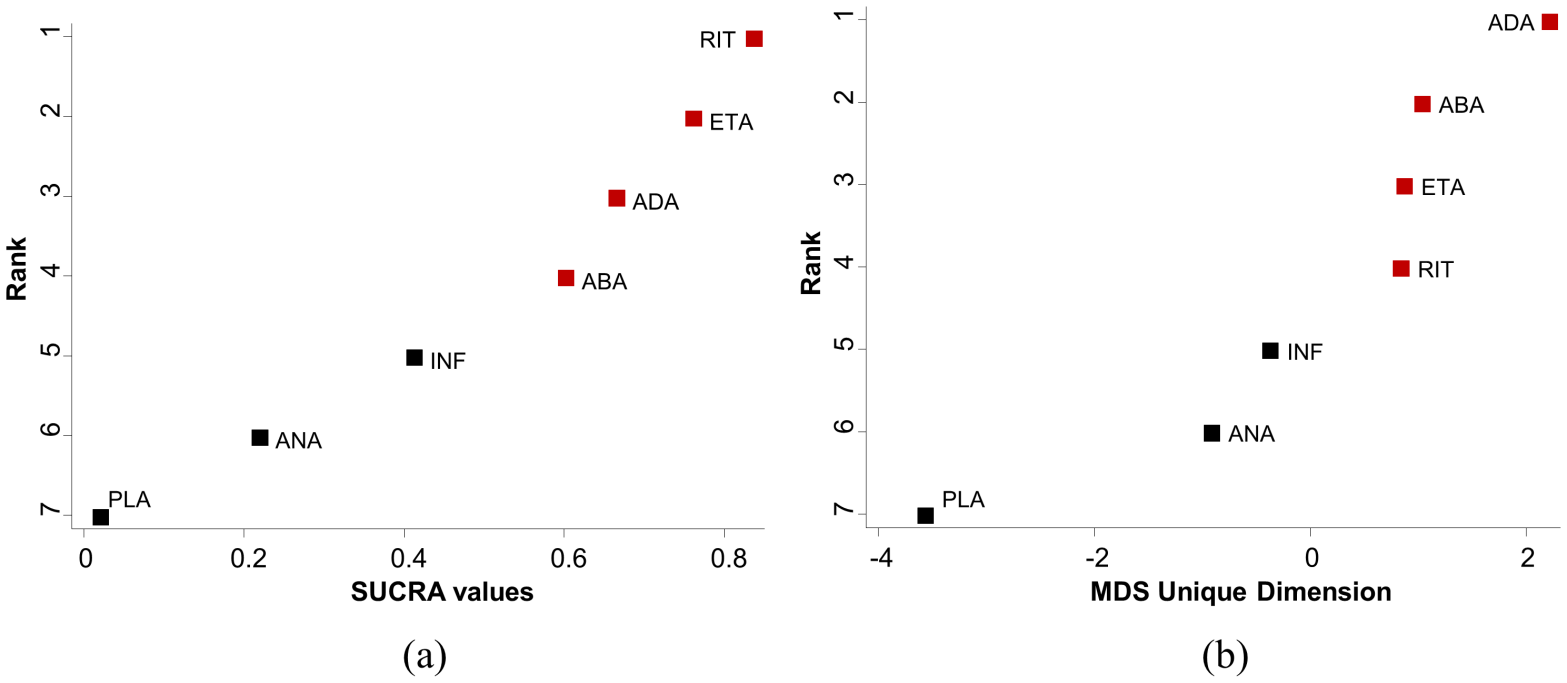
Ranking plots for the rheumatoid arthritis network. Treatments have been ranked (a) according to the surface under the cumulative ranking curves (SUCRA) and (b) according to the unique dimension estimated from multidimensional scaling (MDS) approach. Red points correspond to treatments ranked in different order by the two approaches. (PLA =  placebo, ABA =  abatacept, ADA =  adalimumab, ANA =  anakinra, ETA =  etanercept, INF =  infliximab, RIT =  rituximab).

Figure 8 presents ranking plots for the network of rheumatoid arthritis trials. The vertical axes show the treatments' hierarchy and the horizontal axes the numerical differences using SUCRA values (panel a) and MDS dimension (panel b). There are differences between SUCRA and MDS ranking, however the differences pertains only to the treatments that are close in rank.

The readily-available command mdsmat in STATA performs MDS for a 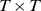 matrix. In case the 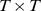 effect sizes' matrix has not been constructed we provide the command mdsrank that constructs the 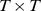 matrix and subsequently calls the mdsmat command. The mdsrank command assumes four arguments; the effect sizes (e.g. lnOR), their standard errors (e.g. selnOR) and the treatments being compared (e.g. t1 t2). MDS finds an underlying dimension of the 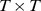 matrix that represents the distances between the *T* treatments and is also used to indicate the ranking of the treatments. The command

. mdsrank lnOR selnOR t1 t2

produces a plot of the underlying dimension showing the ranking of treatments.

The option best(min|max) in mdsrank is used to specify whether smaller or larger values of the estimated unique dimension correspond to better treatment order.

#### 5.3 Clustered ranking plot for two outcomes

Several factors typically need to be taken into account when recommending an intervention, such as its effectiveness, the cost and possible adverse events. Many systematic reviews therefore examine measures of both effectiveness and acceptability, and the ranking of competing treatments for these two outcomes might differ considerably. An appropriate analysis of multiple outcomes should be undertaken using multivariate methods to account for the dependency between outcomes [45], [46]. However, in practice, simultaneous consideration of multiple outcomes for multiple interventions results in cumbersome models and meta-analysts often prefer to analyze each outcome separately. For the case of two outcomes, we recommend the use of two-dimensional plots and clustering methods to obtain meaningful groups of the treatments.

**Figure 9:**
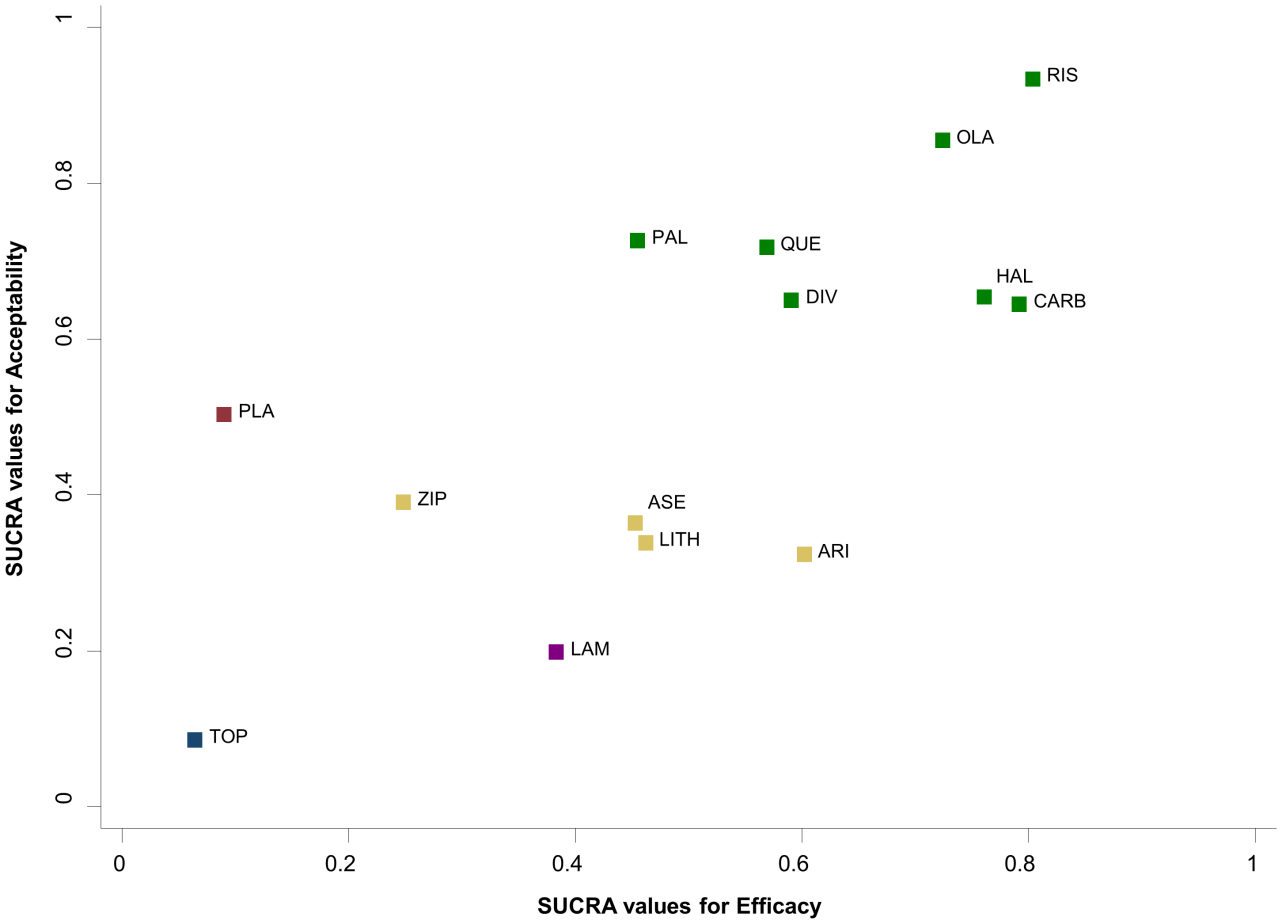
Clustered ranking plot of the acute mania network based on cluster analysis of SUCRA values for two different outcomes: efficacy and acceptability. Each colour represents a group of treatments that belong to the same cluster. Treatments lying in the upper right corner are more effective and acceptable than the other treatments.

Cluster analysis is a common exploratory data mining technique for grouping objects based on their features so that the degree of association is high between members of the same group and low between members of different groups [47]. Figure 9 presents the ranking of the 14 antimanic treatments according to SUCRA values for efficacy and acceptability. The different colors represent the estimated clusters, and are used to group the treatments according to their similarity with regard to both outcomes. For instance, the cluster of treatments on the right upper corner (in green) groups treatments that are acceptable and efficacious. In the Appendix S1 we explain in detail the clustering methods we use to group the treatments and define the optimal number of clusters.

The results of both ranking approaches presented above (SUCRAS and MDS) can be used to produce clusters of treatments for two outcomes. In principle the treatments can be grouped according to more than two outcomes, but the larger the number of outcomes, the more difficult the graphical representation and interpretation of the results.

Clustered ranking plots can be produced in STATA using our clusterank command. Let outcome1 and outcome2 be the data variables containing the SUCRA or MDS scores for all treatments in a network and t be the variable with treatments' codes or names. Then the command

. clusterank outcome1 outcome2 t

returns the cluster ranking plot based on both outcomes.

## Results and Discussion

Although NMA is a useful tool for evidence synthesis, it is often viewed as a complex statistical procedure with many pitfalls. A clear and concise overview of the evidence base and its characteristics, careful consideration of all assumptions and correct interpretation of the findings are crucial but challenging tasks when meta-analyzing data from a network of interventions. In this paper we suggest various graphical tools that can assist researchers interpreting NMA results. We also offer STATA routines to produce these graphical tools. To our knowledge, STATA is the only frequentist software so far where flexible network meta-analysis is possible within a multivariate framework [23], [25]. Our routines can be used jointly with the updated mvmeta command. A recent implementation of NMA in R offers an alternative tool to researchers to apply NMA in a frequentist environment [48].

All graphs presented in this paper address different steps of the analysis. However, the usefulness and interpretation of each graph depends on the nature of the data. For instance, inconsistency plots cannot be used in star-shaped networks, where statistical evaluation of the consistency is untestable. Also, comparison-adjusted funnel plots need a sufficiently large number of studies to judge asymmetry, and clustered ranking plots are not useful when there are only three or four competing treatments. As with any graphical tool, over-interpretation or interpretation in isolation should be avoided and conclusions should be drawn in combination with the numerical results.

## Supporting Information

Figure S1 **Network plot of the coronary artery disease network.** Nodes are weighted according to the number of studies including the respective interventions. Edges are weighted according to the inverse variance of the direct treatment effect estimates for the respective comparisons.
(TIF)

Appendix S1 **Description of the example datasets and script files for the full analysis in STATA.**
(DOC)
