# Supplementary figures and images for: Graphical Tools for Network Meta-Analysis in STATA
Source: PLoS One. 2013 Oct 3;8(10):e76654. doi: 10.1371/journal.pone.0076654 (PMC3789683; doi:10.1371/journal.pone.0076654)

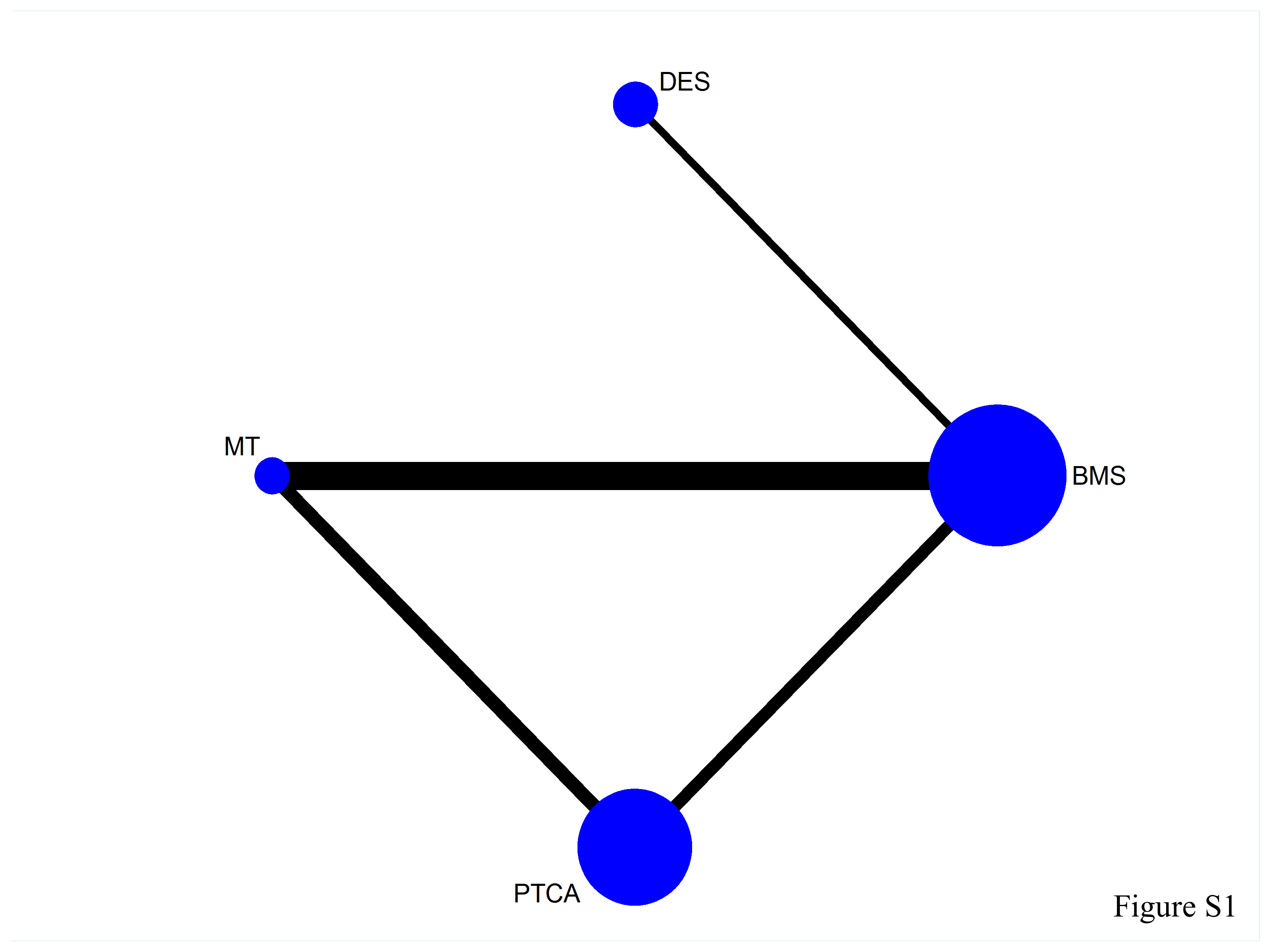

Supplement: Figure S1 — Network plot of the coronary artery disease network. Nodes are weighted according to the number of studies including the respective interventions. Edges are weighted according to the inverse variance of the direct treatment effect estimates for the respective comparisons. (TIF) [file pone.0076654.s001.tif]
