# Supplementary material for: Graphical Tools for Network Meta-Analysis in STATA
Source: PLoS One. 2013 Oct 3;8(10):e76654. doi: 10.1371/journal.pone.0076654 (PMC3789683; doi:10.1371/journal.pone.0076654)
Supplement: Appendix S1 — Description of the example datasets and script files for the full analysis in STATA. (DOC) [file pone.0076654.s002.doc]

**Appendix S1**

# How to install the routines

The installation of all routines requires a live internet connection. Then, with STATA running, the latest versions of the routines can be downloaded by typing in the command window:

For the metan command:

. net install sbe24_3,from(http://www.stata-journal.com/software/sj9-2) replace

For the mvmeta command:

. net install mvmeta,from(http://www.mrc-bsu.cam.ac.uk/IW_Stata/meta) replace

For the metareg command:

. net install sbe23_1,from(http://www.stata-journal.com/software/sj8-4) replace

For the routines that produce the presented graphs:

. net from http://www.mtm.uoi.gr

. net install network_graphs, replace

To see the help files for any routine type help and the name of the command. For example:

. help mvmeta

. help networkplot

Note that the intervalplot, sucra, mdsrank and clusterank commands require running first the mvmeta command, while the commands networkplot, netweight, ifplot and netfunnel can be executed as stand-alone commands.

We illustrate the suggested routines using a series of real data sets, all of which can be downloaded from <http://www.mtm.uoi.gr/example_datasets.rar>.

# Description of the example datasets

## acute mania efficacy pairwise.dta

This network compares the efficacy of 14 pharmacological interventions for acute mania network. It includes 47 studies (36 two-arm and 11 three-arm trials) that provide in total 69 pairwise comparisons (equal to the number of observations). The variable study contains the ID number of each study. The variables t1 and t2, r1 and r2, n1 and n2 contain the name of the treatments (abbreviated), the number of events and the total number of patients in arms 1 and 2. For each multi-arm study with *T* number of arms there are $\frac{T\times(T-1)}{2}$ observations (with the same study ID) equal to the number of pairwise comparisons that this study provides. The variables logOR and selogOR contain the log-odds ratio and its standard error for the comparison t1 vs. t2. Finally, the variable allocation_concealment contains the judgments for the risk of bias regarding the appropriate conduct of allocation concealment (U=unclear, L=low).

## coronary artery disease pairwise.dta

This network includes 62 two-arm trials that evaluate the effectiveness of four different percutaneous coronary interventions for non-acute coronary artery disease. For an explanation of the five variables study, t1, t2, logOR, selogOR see the section above (2.1).

## rheumatoid arthritis pairwise.dta

This is a star-shaped network including 27 placebo-controlled (two-arm) trials that evaluate the effectiveness of six biologic agents for rheumatoid arthritis. For an explanation of the five variables study, t1, t2, logOR, selogOR see the section 2.1.

## acute mania efficacy mvmeta.dta

This is the network of acute mania described in section 2.1, but this dataset includes 47 observations (equal to the total number of studies). The variables t1, t2, t3 contain the ID number of the treatments involved in arms 1, 2 and 3, while for two-arm trials t3 is missing (“.”). Note that treatment 1 corresponds to placebo and we have coded all other competing treatments in the numerical order (2 to 14) that corresponds to the alphabetical order of their names. The variables r1-r14 and n1-n14 contain the number of events and the total number of patients for each study treatment. We considered placebo as the reference intervention and we replaced the missing values in r1 and n1 with the values 0.001 and 0.01 respectively (see [1,2] for details on data augmentation). The variables y2-y14 and S22-S1414 contain the log-odds ratios of each treatment vs. the reference (placebo) and their variances. Finally, the variables S23-S1314 contain the covariances between two log-odds ratios in multi-arm studies.

## acute mania acceptability mvmeta.dta

This network compares 15 pharmacological interventions for acute mania regarding their acceptability and includes 64 studies (46 two-arm and 18 three-arm trials). For an explanation of the variables r1-r15, n1-n15, y2-y15, S22-S1515 see the section above (2.4).

## rheumatoid arthritis mvmeta.dta

This network is the network of rheumatoid arthritis described in section 2.3. For an explanation of the variables r1-r7, n1-n7, y2-y7, S22-S77 see the section 2.4.

## rheumatoid arthritis lnOR estimates.dta

This dataset includes all the pairwise log-odds ratio estimates of the rheumatoid arthritis network (described in section 2.3) derived after performing network meta-analysis with the mvmeta command. The variables t1 and t2 contain the names of the treatments (abbreviated) involved in each pairwise comparison. The variables lnOR and selnOR contain the network estimates for each comparison t1 vs. t2.

## acute mania SUCRA values.dta

This dataset includes the SUCRA values for the efficacy and acceptability outcomes of the acute mania network (described in sections 2.1 and 2.5). To derive these SUCRA values (stored in variables efficacy and acceptability) we used the ranking probabilities estimated from the mvmeta command. The variable treatment contains the names of the treatments (abbreviated) that correspond to each pair of SUCRA values.

# How to produce the network plot

The networkplot command requires a dataset in which each row corresponds to a pairwise comparison between two treatments; hence, for multi-arm trials all pairwise comparisons need to be given. For example, if study *i* compares $T_{i}$ treatments, all $\left(\begin{matrix} T_{i} \\ 2 \end{matrix}\right)$ possible pairwise comparisons need to be given. The same type of dataset is appropriate also for the commands netweight, ifplot and netfunnel.

To open the dataset of the acute mania network for the efficacy outcome that contains all possible pairwise comparisons, type (after specifying the working directory):

. use "acute mania efficacy pairwise.dta", replace

Then, typing:

. networkplot t1 t2

produces a network plot in which both nodes and edges are weighted according to the number of studies evaluating each treatment and direct comparison respectively.

To produce the network plot of Figure 1 where the width of the edges is proportional to the mean control group risk for all comparisons versus placebo, first calculate the control group risk for studies including the placebo:

. gen cgr=r1/n1 if t1=="PLA"

(51 missing values generated)

. replace cgr=r2/n2 if t2=="PLA"

(29 real changes made)

and then type:

. networkplot t1 t2, edgew(cgr mean)

The full names of the treatments can be displayed in the plot with the option labels() as follows:

. networkplot t1 t2, edgew(cgr mean) lab(Aripiprazole Asenapine Carbamazpine Divalproex Haloperidol Lamotrigine Lithium Olanzapine Paliperidone Placebo Quetipaine Ripseridone Topiramate Ziprasidone) asp(0.8) edgesc(2.5)

Note that the names of the treatments need to be given in alphabetical order. The option asp() changes the aspect ratio of the plot and the option edgescale() is used to increase the thickness of all edges and make the plot more legible.

To color the edges according to the adequacy of allocation concealment in the majority of trials (Figure 2) type:

. networkplot t1 t2, edgecol(by allocation_concealment) lab(Aripiprazole Asenapine Carbamazpine Divalproex Haloperidol Lamotrigine Lithium Olanzapine Paliperidone Placebo Quetipaine Ripseridone Topiramate Ziprasidone) asp(0.8)

Note that here we use the default weights for both nodes and edges. However, the weighting and coloring options can be combined.

# How to produce the contribution plot

To open the dataset of the coronary artery disease network that contains all the pairwise comparisons, type:

. use "coronary artery disease pairwise.dta", replace

The contribution plot can be produced by typing:

. netweight logOR selogOR t1 t2,asp(0.8) sc(0.9)

The command gives also the following output results:

Direct comparisons and number of included studies:

BMSvsDES 16

BMSvsMT 3

BMSvsPTCA 33

MTvsPTCA 10

Indirect comparisons:

DESvsMT

DESvsPTCA

Y[4,1]

-0.080

-0.041

-0.087

0.165

symmetric V[6,6]

.0425 0 0 0 0 0

0 .0123 0 0 0 0

0 0 .027 0 0 0

0 0 0 .0253 0 0

0 0 0 0 10000 0

0 0 0 0 0 10000

Note: Effect sizes of 36 observations were reversed (stored in matrix e(R))

Basic contrasts:

BMSvsDES BMSvsMT BMSvsPTCA

Design matrix:

X[6,3]

1 0 0

0 1 0

0 0 1

0 -1 1

-1 1 0

-1 0 1

Contribution of each direct comparison in each pairwise summary effect:

H[6,4]

1.000 0.000 0.000 0.000

0.000 0.810 0.190 -0.190

0.000 0.418 0.582 0.418

0.000 -0.392 0.392 0.608

-1.000 0.810 0.190 -0.190

-1.000 0.418 0.582 0.418

Percentage contribution of each direct comparison in each pairwise summary effect:

P[6,4]

100.0 0.0 0.0 0.0

0.0 68.0 16.0 16.0

0.0 29.5 41.0 29.5

0.0 28.1 28.1 43.7

45.7 37.0 8.7 8.7

41.4 17.3 24.1 17.3

Percentage contribution of each direct comparison in the entire network:

C[1,4]

31.2 29.6 20.2 19.0

# How to produce the inconsistency plot

To open the dataset of the acute mania network for the efficacy outcome that contains all the pairwise comparisons, type:

. use "acute mania efficacy pairwise.dta", replace

Then, the inconsistency plot can be produced by typing:

. ifplot logOR selogOR t1 t2 study,plotopt(classic texts(150)) eform xlab(2,5,20,50)

Note that we assume within each loop a common heterogeneity estimated by the method of moments estimator. Other options are also available (see the help file for more details).

In the option plotoptions() we use options allowed in the metan command for the appearance of the forest plot. With the addition of the option eform the inconsistency plot includes ratio of odds ratios (*RoR*s) instead of the absolute inconsistency factors.

The command gives also the following output results:

* 17 triangular loops found

* 4 quadratic loops found

Note: Heterogeneity of loop HAL-OLA-RIS cannot be estimated due to insufficient observations - set equal to 0

Evaluation of inconsistency using loop-specific heterogeneity estimates:

+---------------------------------------------------------------------------------+

| Loop | RoR | z_value | p_value | CI_95 | Loop_Heterog_tau2 |

|------------------+-------+---------+---------+--------------+-------------------|

| CARB-DIV-PLA | 3.448 | 1.260 | 0.208 | (1.00,23.62) | 0.076 |

| CARB-DIV-HAL-OLA | 2.947 | 0.808 | 0.419 | (1.00,40.58) | 0.053 |

| ARI-HAL-LITH-QUE | 2.624 | 1.286 | 0.199 | (1.00,11.41) | 0.101 |

| ARI-HAL-LITH-OLA | 2.058 | 0.919 | 0.358 | (1.00,9.60) | 0.091 |

| LITH-OLA-PLA | 1.923 | 1.170 | 0.242 | (1.00,5.75) | 0.091 |

| LITH-PLA-QUE | 1.713 | 1.472 | 0.141 | (1.00,3.51) | 0.038 |

| CARB-HAL-PLA | 1.704 | 0.478 | 0.633 | (1.00,15.13) | 0.105 |

| ASE-OLA-PLA | 1.690 | 1.059 | 0.290 | (1.00,4.46) | 0.049 |

| HAL-PLA-QUE | 1.620 | 1.143 | 0.253 | (1.00,3.70) | 0.046 |

| PAL-PLA-QUE | 1.607 | 1.049 | 0.294 | (1.00,3.90) | 0.071 |

| DIV-LITH-OLA | 1.596 | 0.473 | 0.637 | (1.00,11.10) | 0.195 |

| OLA-PLA-RIS | 1.585 | 1.004 | 0.315 | (1.00,3.90) | 0.094 |

| HAL-OLA-RIS | 1.426 | 0.943 | 0.346 | (1.00,2.98) | 0.000 |

| DIV-OLA-PLA | 1.416 | 0.934 | 0.350 | (1.00,2.94) | 0.077 |

| ARI-HAL-PLA | 1.416 | 1.025 | 0.305 | (1.00,2.75) | 0.067 |

| ARI-LITH-PLA | 1.384 | 0.724 | 0.469 | (1.00,3.34) | 0.070 |

| HAL-LITH-OLA-QUE | 1.323 | 0.284 | 0.776 | (1.00,9.14) | 0.208 |

| HAL-PLA-ZIP | 1.225 | 0.465 | 0.642 | (1.00,2.88) | 0.074 |

| DIV-LITH-PLA | 1.147 | 0.210 | 0.834 | (1.00,4.13) | 0.107 |

| HAL-OLA-PLA | 1.070 | 0.176 | 0.860 | (1.00,2.28) | 0.079 |

| HAL-PLA-RIS | 1.042 | 0.071 | 0.943 | (1.00,3.21) | 0.153 |

+---------------------------------------------------------------------------------+

# How to produce the ‘comparison-adjusted’ funnel plot

To open the dataset of the rheumatoid arthritis network that contains all the pairwise comparisons, type:

. use "rheumatoid arthritis pairwise.dta", replace

Then, the comparison-adjusted funnel plot can be produced by typing:

. netfunnel logOR selogOR t1 t2,bycomp add(lfit selogOR _ES_CEN) noalpha

With the option bycomparison we specify that different colors are used for the different comparisons, while the option noalphabetical plots the comparisons t1 vs. t2 as these have been specified in the dataset for all studies irrespectively of their alphabetical order.

The command adds in the dataset the new variable _ES_CEN that contains for each study the difference between the observed effect size and comparison-specific summary effect (). Using the option addplot(), we regress this variable on standard error and add in the plot a simple linear regression line to help us explore visually if there is a systematic bias in the results between small and large studies.

The command gives also the available comparisons as output results:

Comparisons in the plot:

1. PLA vs RIT

2. PLA vs INF

3. PLA vs ETA

4. PLA vs ANA

5. PLA vs ADA

6. PLA vs ABA

# How to produce the predictive interval plot

To produce the predictive interval plot, the first step is to perform network meta-analysis using the mvmeta command.

To open the dataset of the rheumatoid arthritis network suitable to run the mvmeta command, type:

. use "rheumatoid arthritis mvmeta.dta", replace

and perform network meta-analysis:

. mat P = I(6) + J(6,6,1)

. mvmeta y S, bscov(prop P)

Note that we assume a common heterogeneity estimate for all comparisons (with the bscov()option).

Then, the predictive interval plot can be produced by typing:

. intervalplot,mvmeta pred eform lab(PLA ABA ADA ANA ETA INF RIT) null(1) sep marg(10 40 2 2)

The option predictions specifies the inclusion of the predictive intervals (along with the confidence intervals) in the plot and the option eform specifies that odds ratios are plotted instead of log-odds ratios.

With the option null()and labels() we add the line of no effect and we specify the names of the treatment (in numerical order).

Finally, with the options separate and margin() we plot the treatment effects in subgroups according to the comparator treatment and we handle the margins for the region of the plot.

# How to produce ranking plots for a single outcome using probabilities

To produce the ranking plots, the first step is to perform network meta-analysis using the mvmeta command and to estimate the ranking probabilities for all competing treatments.

To open the dataset of the rheumatoid arthritis network suitable to run the mvmeta command, type:

. use "rheumatoid arthritis mvmeta.dta", replace

Then, the ranking probabilities can be estimated using the pbest()option in the mvmeta command as follows:

. mat P = I(6) + J(6,6,1)

. mvmeta y S, bscov(prop P) pbest(max,all zero gen(prob) reps(50000))

With the sub-option max we specify that the outcome of the network is beneficial, the all and zero options specify the derivation of probabilities for all possible ranks for each treatment (with the exception of the reference treatment) and for the reference treatment (i.e. placebo) respectively.

We also ask for 50000 draws of the linear predictors in the option reps()(see the help file for more details). Note that the mvmeta command takes several minutes to run when we add the option reps(50000). To reduce the required time we can ask for fewer draws (e.g. 10000) or omit the option and use the default number of draws (1000).

The variables probi_j that are added to the dataset contain the probability that treatment *j* is at the *i^th^* order.

To derive the ranking probabilities for the adjusted for small-study effects model, we need first to create the variances of the observed log-odds ratios:

foreach var in S22 S33 S44 S55 S66 S77{

gen var_`var'=`var'

replace var_`var'=0 if var_`var'==.

}

Then the ranking probabilities for the meta-regression model, that uses these variances as covariates, can be derived by typing:

. mvmeta y S var*, bscov(prop P) pbest(max,all zero reps(50000))

However, when the model includes covariates the mvmeta estimates a different probability for each study with respect to the different values of the covariates. Thus, we need before estimating the ranking probabilities to ‘impute’ a hypothetical study with minimal information for all treatments and the desired value for the covariates (the variance in this example) as follows:

. set obs 28

forvalues i=1/7{

replace r`i’=0.001 in 28

replace n`i’=0.01 in 28

}

foreach trt in 1 2 3 4 5 6 7{

if `trt'==1 continue

replace y`trt' in 28=log(r`trt'/(n`trt'-r`trt'))-log(r1/(n1-r1))

replace S`trt'`trt' in 28=1/r`trt'+1/(n`trt'-r`trt')+1/r1+1/(n1-r1)

foreach trt2 in 1 2 3 4 5 6 7{

if `trt2'==1 continue

if `trt2'>`trt' replace S`trt'`trt2' in 28=1/r1+1/(n1-r1) if r`trt'!=. & n`trt2'!=.

}

}

Then, for the imputed study we set the covariates (var_S22, var_S33, …) equal to the variance of the largest study:

forvalues i=2/7{

forvalues j=1/27{

replace var_S`i’`i’ in 28=max(var_S`i’`i’[28], var_S`i’`i’[`j’])

}

}

and we estimate the ranking probabilities using this dataset:

. mvmeta y S, bscov(prop P) pbest(max,all zero gen(sseprob) reps(50000)) eq(y2:var_S22,y3:var_S33,y4:var_S44,y5:var_S55, y6:var_S66,y7:var_S77)

foreach var of varlist sse*{

replace `var’=`var’[28]

}

. drop in 28

The two sets of ranking probabilities can be used to produce the cumulative ranking curve plots for all treatments in the same plot, as follows:

. sucra prob*,mvmeta comp(sseprob*) lab(Placebo Abatacept Adalimumab Anakinra Etanercept Infliximab Rituximab) names("Model without adjustment" "Model adjusted for small-study effects")

Note that the names of treatments in the option lab() need to be specified in numerical order. In the option names() we specify the names of the two different models that are displayed in the plot.

The command gives also as output results the SUCRA values (numerical summaries of the surface under the cumulative ranking curves) and the probability for each treatment to be the best:

# Treatment Relative Ranking of Model without adjustment

# +-----------------------------+

# | Treatment | SUCRA | PrBest |

# |------------+-------+--------|

# | Placebo | 1.8 | 0.0 |

# | Abatacept | 59.8 | 7.6 |

# | Adalimumab | 66.1 | 9.4 |

# | Anakinra | 22.0 | 0.3 |

# | Etanercept | 76.2 | 30.1 |

# | Infliximab | 40.8 | 3.4 |

# | Rituximab | 83.4 | 49.3 |

# +-----------------------------+

# Treatment Relative Ranking of Model adjusted for small-study effects

# +-----------------------------+

# | Treatment | SUCRA | PrBest |

# |------------+-------+--------|

# | Placebo | 2.9 | 0.0 |

# | Abatacept | 34.1 | 1.7 |

# | Adalimumab | 61.2 | 3.1 |

# | Anakinra | 51.4 | 5.6 |

# | Etanercept | 92.0 | 74.3 |

# | Infliximab | 57.5 | 11.7 |

# | Rituximab | 50.8 | 3.5 |

# +-----------------------------+

# How to produce ranking plot for a single outcome using multidimensional scaling

To open the dataset of the rheumatoid arthritis network suitable to run the mvmeta command, type:

. use "rheumatoid arthritis mvmeta.dta", replace

After performing a network meta-analysis all pairwise summary effect estimates can be derived by typing:

. mat P = I(6) + J(6,6,1)

. mvmeta y S, bscov(prop P)

foreach trt1 in 2 3 4 5 6 7{

foreach trt2 in 2 3 4 5 6 7{

if `trt1'==`trt2' continue

if `trt2'>`trt1' lincom y`trt2'-y`trt1'

}

}

We have stored all these pairwise summary effects in a dataset.

To open the dataset including all the summary pairwise ln(OR)s with their standard errors for rheumatoid arthritis network, type:

. use "rheumatoid athritis lnOR estimates.dta", replace

Then, the ranking plot based on multidimensional scaling (MDS) can be produced using the following command:

. mdsrank lnOR selnOR t1 t2

The command gives also as output results the MDS scores and the respective rank:

+-------------------------+

| Treatm~t | Dim1 | Rank |

|----------+-------+------|

| PLA | -3.59 | 1 |

| ANA | -0.93 | 2 |

| INF | -0.38 | 3 |

| RIT | 0.82 | 4 |

| ETA | 0.86 | 5 |

| ABA | 1.02 | 6 |

| ADA | 2.20 | 7 |

+-------------------------+

Note that the treatment order is the opposite than the expected order (placebo is first). The mdsrank command, by default, assumes that lower MDS scores correspond to more effective treatments. However, this is not the case as in our example (the method requires some prior knowledge on the results).

We run again the command and we specify in the option best() that larger MDS score correspond to more effective treatments:

. mdsrank lnOR selnOR t1 t2, best(max) scat(msymb(square))

and we get the following treatment order:

+-------------------------+

| Treatm~t | Dim1 | Rank |

|----------+-------+------|

| ADA | 2.20 | 1 |

| ABA | 1.02 | 2 |

| ETA | 0.86 | 3 |

| RIT | 0.82 | 4 |

| INF | -0.38 | 5 |

| ANA | -0.93 | 6 |

| PLA | -3.59 | 7 |

+-------------------------+

In the option scatteroptions() we use options allowed in the scatter command to handle the appearance of the plot.

# How to produce clustered ranking plot for two outcomes

For the acute mania network we have available data for two outcomes, efficacy and acceptability. Analyzing each outcome separately we can get for each one the respective treatment order as follows:

To open the dataset of the acute mania network for the efficacy outcome suitable to run the mvmeta command, type:

. use "acute mania data efficacy mvmeta.dta", replace

and perform network meta-analysis estimating the ranking probabilities:

. mat P = I(13) + J(13,13,1)

. mvmeta y S, bscov(prop P) pbest(max,all zero gen(prob) reps(50000))

Then, using the sucra command we can calculate the SUCRA values for all treatments:

. sucra prob*,mvmeta lab(Placebo Aripiprazole Asenapine Carbamazpine Divalproex Haloperidol Lamotrigine Lithium Olanzapine Paliperidone Quetipaine Ripseridone Topiramate Ziprasidone) noplot

+-------------------------------+

| Treatment | SUCRA | PrBest |

|--------------+-------+--------|

| Placebo | 9.0 | 0.0 |

| Aripiprazole | 45.3 | 5.0 |

| Asenapine | 79.2 | 36.7 |

| Carbamazpine | 59.1 | 2.7 |

| Divalproex | 76.1 | 8.9 |

| Haloperidol | 38.3 | 17.2 |

| Lamotrigine | 46.2 | 0.8 |

| Lithium | 72.4 | 3.8 |

| Olanzapine | 60.2 | 1.8 |

| Paliperidone | 45.5 | 2.4 |

| Quetipaine | 56.9 | 1.5 |

| Ripseridone | 80.4 | 19.0 |

| Topiramate | 6.4 | 0.1 |

| Ziprasidone | 24.8 | 0.0 |

+-------------------------------+

The same way we can get the treatment order for the acceptability outcome:

. use "acute mania data acceptability mvmeta.dta", replace

. mat P = I(14) + J(14,14,1)

. mvmeta y S, bscov(prop P) pbest(max,all zero gen(prob) reps(50000))

. sucra prob*,mvmeta lab(Placebo Aripiprazole Asenapine Carbamazpine Gabapentin Divalproex Haloperidol Lamotrigine Lithium Olanzapine Paliperidone Quetipaine Ripseridone Topiramate Ziprasidone) noplot

+-------------------------------+

| Treatment | SUCRA | PrBest |

|--------------+-------+--------|

| Placebo | 32.4 | 0.0 |

| Aripiprazole | 36.4 | 0.2 |

| Asenapine | 64.5 | 14.3 |

| Carbamazpine | 65.0 | 1.9 |

| Gabapentin | 11.2 | 0.4 |

| Divalproex | 65.4 | 1.1 |

| Haloperidol | 19.9 | 0.1 |

| Lamotrigine | 33.9 | 0.0 |

| Lithium | 85.5 | 13.5 |

| Olanzapine | 50.3 | 0.1 |

| Paliperidone | 72.6 | 11.8 |

| Quetipaine | 71.8 | 3.1 |

| Ripseridone | 93.4 | 53.5 |

| Topiramate | 8.6 | 0.0 |

| Ziprasidone | 39.1 | 0.0 |

+-------------------------------+

We have stored the two sets of SUCRA values in a dataset.

To open the dataset that contains the SUCRA values of the acute mania network for the efficacy and acceptability outcomes type:

. use "acute mania sucra values.dta", replace

Then, the clustered ranking plot can be derived by typing:

Note that the acceptability outcome includes one more treatment than the efficacy outcome. However, the clusterank command requires for all treatments results on both outcomes to perform cluster analysis. Thus, we use only the 14 treatments with results on both outcomes.

## Description of the undertaken cluster analysis. To group the competing treatments of a network we use a hierarchical agglomerative clustering method evaluating different metrics (Euclidean, squared Euclidean, absolute-value distance, etc.) and linkage methods (single, average, weighted, complete, ward, centroid, median) [3]. The choice of the appropriate metric and linkage criterion is driven from the cophenetic correlation coefficient, which measures how faithfully the output dendrogram represents the dissimilarities among observations [4]. To choose the optimal level of dendrogram and define the optimal number of resulting partitions, we used an internal cluster validation measure based on a value of ‘clustering gain’ that has been designed to have a maximum value when intra-cluster similarity is maximized and inter-cluster similarity is minimized [5].

The command gives also the following output results:

Best linkage method: singlelinkage

Best distance metric: Canberra

Cophenetic Correlation Coefficient c = 0.92

** Maximum value of clustering gain = 5468.80

** Optimal number of clusters = 5

References

1. White IR, Barrett JK, Jackson D, Higgins JPT (2012) Consistency and inconsistency in network meta-analsyis: model estimation using multivariate meta-regression. Res Syn Meth 3: 111-125.

2. White IR (2011) Multivariate random-effects meta-regression: Updates to mvmeta. The STATA Journal 11: 255-270.

3. Kaufman, L. and Rousseeuw, P. J. (1990) Finding groups in data: An introduction to cluster analysis. New York: Wiley.

4. Handl J, Knowles J, Kell DB (2005) Computational cluster validation in post-genomic data analysis. Bioinformatics 21: 3201-3212.

5. Jung Y, Park H, Du D (2003) A decision criterion for the optimal number of clusters in hierarchical clustering. J Global Optimazation 25: 91-111.
